# Genomic Epidemiology and Antimicrobial Resistance Mechanisms of Imported Typhoid in Australia

**DOI:** 10.1128/AAC.01200-21

**Published:** 2021-11-17

**Authors:** Danielle J. Ingle, Patiyan Andersson, Mary Valcanis, Mathilda Wilmot, Marion Easton, Courtney Lane, Jessica Barden, Anders Gonçalves da Silva, Torsten Seemann, Kristy Horan, Susan A. Ballard, Norelle L. Sherry, Deborah A. Williamson, Benjamin P. Howden

**Affiliations:** a Department of Microbiology and Immunology at the Peter Doherty Institute for Infection and Immunity, The University of Melbourne, Melbourne, Australia; b Microbiological Diagnostic Unit Public Health Laboratory, Department of Microbiology and Immunology at the Peter Doherty Institute for Infection and Immunity, The University of Melbourne, Melbourne, Australia; c Department of Health, Victoria, Australia; d Department of Infectious Diseases, Austin Health, Melbourne, Australia; e Royal Melbourne Hospital at the Peter Doherty Institute for Infection and Immunity, Melbourne, Australia; f Doherty Applied Microbial Genomics, Department of Microbiology and Immunology at the Peter Doherty Institute for Infection and Immunity, The University of Melbourne, Melbourne, Australia

**Keywords:** antimicrobial resistance, genomics, typhoid

## Abstract

Typhoid fever is an invasive bacterial disease of humans that disproportionately affects low- and middle-income countries. Antimicrobial resistance (AMR) has been increasingly prevalent in recent decades in Salmonella enterica serovar Typhi (S. Typhi), the causative agent of typhoid fever, limiting treatment options. In Australia, most cases of typhoid fever are imported due to travel to regions where typhoid fever is endemic. Here, all 116 isolates of S. Typhi isolated in Victoria, Australia, between 1 July 2018 and 30 June 2020, underwent whole-genome sequencing and antimicrobial susceptibility testing. Genomic data were linked to international travel data collected from routine case interviews. Travel to South Asia accounted for most cases, with 92.2% imported from seven primary countries (the top two were India, *n* = 87, and Pakistan, *n* = 12). A total of 17 S. Typhi genotypes were detected in the 2-year cohort, with 48.2% genotyped as part of global AMR lineages. Ciprofloxacin resistance was detected in two lineages, 3.3 and 4.3.1.2, all from cases with reported travel to India. Nearly all multidrug and extensively drug resistant isolates (90%) were from cases with reported travel to Pakistan in genotypes 4.3.1.1 and 4.3.1.1.P1. Extended spectrum beta-lactamases, *bla*CTX-M-15 and *bla*SHV-12, were detected in cases with travel to Pakistan and India, respectively. Linking epidemiological data with genomic studies of S. Typhi provides an opportunity to improve understanding of the emergence, spread and risk of drug-resistant S. Typhi infections and to better inform empirical treatment guidelines in returned travelers.

## INTRODUCTION

The prevention, treatment, and control of typhoid fever remains a significant public health challenge in the 21st century (1). Salmonella enterica serovar Typhi (S. Typhi) is the causative agent of typhoid fever (2) and is estimated to cause 10.9 million infections and 116,800 deaths globally each year (3). This burden of disease disproportionately affects children < 5 years in low-middle income countries (2, 3). In high income countries, cases of typhoid fever most commonly occur as a result of recent international travel to regions where S. Typhi is endemic (4–6). Antimicrobial therapy is the mainstay of treatment for typhoid fever, however successive waves of antimicrobial resistance (AMR) in S. Typhi raises the specter of untreatable typhoid fever, especially with oral antimicrobials (1, 5). Recently, genomic studies have started to provide critical insights into the international spread and mechanisms of antimicrobial resistance in Salmonella Typhi (6–15).

Multidrug resistance (MDR) to the initial first line drugs for treating typhoid fever, ampicillin, co-trimoxazole, and chloramphenicol, first appeared in the 1960s (5). AMR was a significant driver in the global dissemination of the lineage 4.3.1 (haplotype 58) soon after it emerged in the late 1980s/early 1990s (8, 16). Initially MDR was associated with IncHI1 plasmids and has been detected globally (8, 10, 17). The subsequent integration of a MDR composite transposon into the chromosome at different IS*1* sites is a key feature of sublineage 4.3.1.1 (8).

Resistance to fluoroquinolones emerged in the late 1990s (5). A hallmark of the second sublineage of the global clone, 4.3.1.2, are triple-point mutations in quinolone resistance determining regions (QRDRs) that confer resistance to ciprofloxacin and has been associated with fluoroquinolone treatment failure in South Asia (18, 19). Reduced susceptibility to ciprofloxacin results from single- or double-point mutations in QRDR (19). These point mutations have limited to no fitness cost and so are maintained in populations even once the selective pressure of fluoroquinolone use has ceased (19, 20).

Alarmingly, resistance to extended spectrum beta-lactams and azithromycin has been reported recently in S. Typhi (4, 14, 21–23). Extended spectrum beta-lactamases (ESBLs) have been largely associated with mobilization on IncY plasmids, although the recent study of Nair et al. showed different types of chromosomal integration of *bla*CTX-M genes (4). A new sublineage of extensively drug resistant (XDR) S. Typhi, 4.3.1.1.P1, emerged from Pakistan (14). This XDR threat is a sublineage of the MDR 4.3.1.1 that has acquired an IncY plasmid carrying *bla*CTX-M-15 and *qnrS1* genes and together with the single QRDR point mutation, *gyrA*-S83F, these confer resistance to ESBLs and ciprofloxacin (4, 14). Azithromycin is the only remaining oral therapeutic option for these XDR S. Typhi (5). Further, point mutations in the *acrB* gene have been shown to confer resistance to azithromycin, the last remaining oral therapeutic that is broadly efficacious in South Asia for typhoid fever (12, 22–24). These new resistance profiles are associated with South Asia, however S. Typhi isolates resistant to extended spectrum beta-lactams or azithromycin have been detected in returned travelers from these regions (4, 15, 25).

In Australia, typhoid fever is a nationally notifiable disease, and vaccination is recommended for travelers to regions where typhoid is prevalent (26). Moreover, as almost all cases of typhoid in Australia are acquired overseas, the appropriateness of empirical therapy is dependent on the resistance profiles of S. Typhi in the region where the infection was acquired. The increasing prevalence of XDR and azithromycin resistant cases of typhoid fever in South Asia highlights the need for enhanced genomic surveillance of S. Typhi globally. Here, to better understand the genomic epidemiology and resistance determinants of imported S. Typhi we undertook a 2-year study of S. Typhi cases reported in Victoria, Australia. Our results inform approaches for optimizing genomic surveillance of S. Typhi and empirical treatment approaches based on region of travel.

## RESULTS

### Comprehensive 2-year cohort.

**FIG 1:**
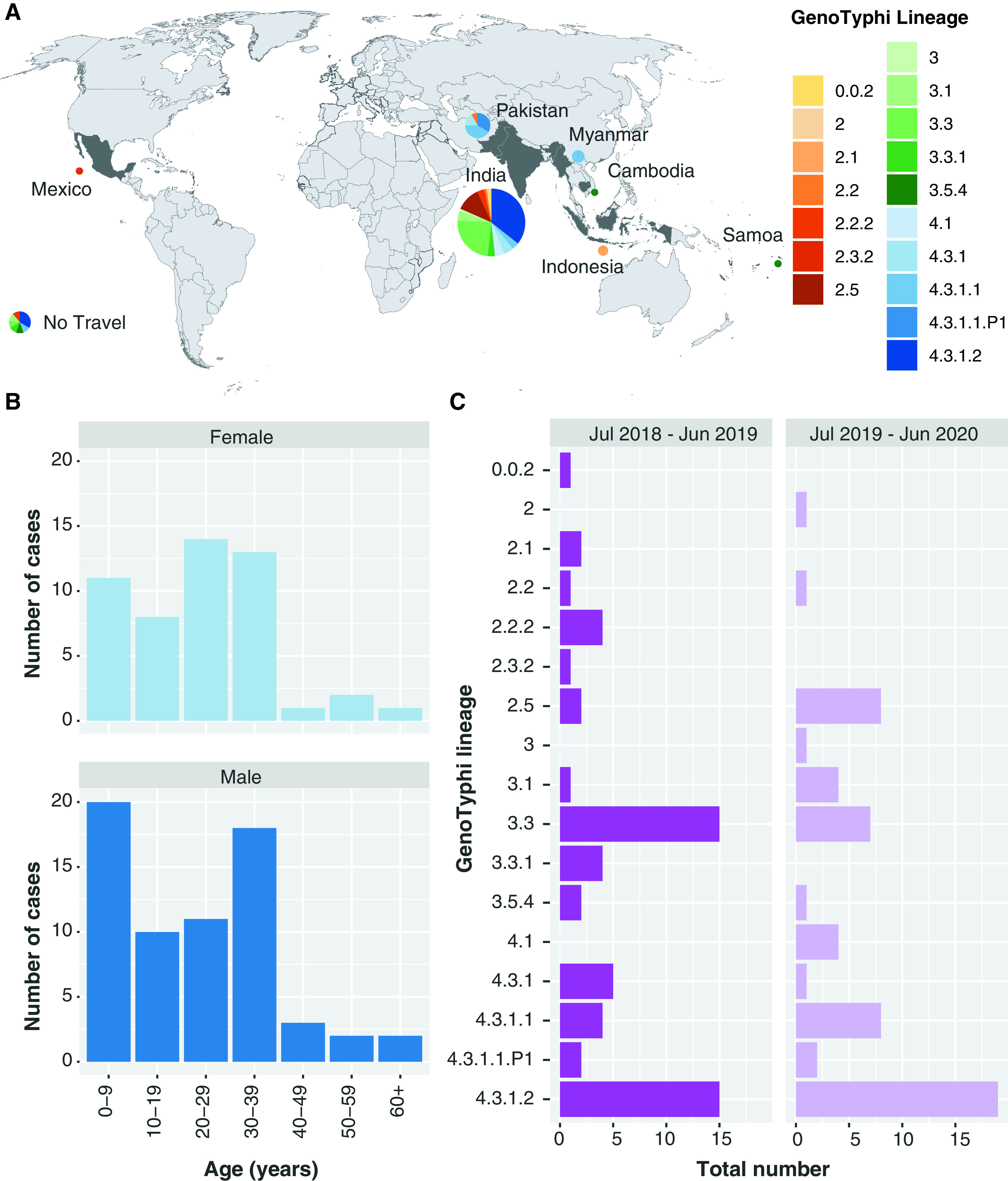
Summary of the 116 Salmonella enterica serovar Typhi (S. Typhi) isolates from the 2-year study period. A: Distribution of 116 S. Typhi isolates with reported international travel. Pie graphs represent the proportion of isolates with reported travel to different countries or no reported travel. The graphs are colored by membership to GenoTyphi lineages. Blank map sourced from https://commons.wikimedia.org/wiki/File:BlankMap-World-Flattened.svg. B: Patient characteristics of individuals which the S. Typhi were isolated. The histograms show number of cases of male and female patients, stratified by age (years). C: Membership to the different GenoTyphi lineages over the 2-year study period.

Between 1 July 2018 and 30 June 2020, a total of 116 S. Typhi isolates were received at the state reference laboratory, MDU PHL in Victoria, Australia (Fig. 1). The 116 S. Typhi were subject to routine whole-genome sequencing (WGS), which commenced for all serovars of Salmonella enterica from 1 July 2018. The number of cases was consistent between 2 years (first timespan, 1 July 2018 to 30 June 2019, *n* = 57; second timespan, 1 July 2019 to 30 June 2020, *n* = 59). These data represent a sampling fraction of 96.7% of the S. Typhi cases notified in Victoria, Australia, over the 2-year study period and provide an unbiased and comprehensive cohort of the S. Typhi causing infections.

### *Genotypes and epidemiological characteristics of* S. Typhi.

The S. Typhi isolates from the 116 cases of typhoid fever were assigned to one of 17 genotypes using GenoTyphi (8, 24), and linked to the reported international travel available for each of the patients (Fig. 1A, Fig. S1c). Travel was reported to seven countries with the vast majority reporting travel to India (87/116, 75.0%). Of the 87 cases with reported travel to India, the most common genotypes were 4.3.1.2 (*n* = 31), 3.3 (*n* = 21) and 2.5 (*n* = 10) (Table S1). Pakistan was the next most frequent destination for travelers (12/116, 10.4%) with the majority of isolates part of the global sublineages 4.3.1.1 (*n* = 5) or 4.3.1.1.P1 (*n* = 4). Fewer than five cases were associated with reported travel to each of Samoa, Cambodia, Mexico, Myanmar and Indonesia. No travel was reported for 9/116 (7.8%) although one case had confirmed contact with a returned traveler from India and another, AUSMDU00019653, confirmed contact with a chronic carrier.

Epidemiological data including age, sex and date of sample collection was available for all 116 cases (Fig. 1B and C). The proportion of cases from males was slightly higher than from females (66/116, 56.9%), however this difference was not significant (*P* = 0.16, two-sided test of proportions). The number of cases from males and females was consistent over the 2 years (*n* = 25 from females each 12-month period and *n* = 34 and *n* = 32 from males in the first and second timespan, respectively). The median age of all cases was 25 years (interquartile range [IQR] 8-32 years. This was consistent between males and females with the median age for males being 24 years (IQR 7.25–33) and for females being 27 years (IQR 10–31). There were some differences in the most common genotypes detected between the two timespans (Fig. 1C). Lineage 4.3.1.2 was the most common in both sampling frames, however lineage 3.3 (associated with returned travelers from India) decreased in prevalence while lineage 2.5 increased (also associated with returned travelers from India).

Investigation of the isolate from the chronic carrier, AUSMDU00017205, and the epidemiologically linked case, AUSMDU00019653, found little genomic difference between the two isolates. The isolate from the chronic carrier was received in early June 2018 with the linked case received 4 months later in September 2018. Both were genotyped as 2.2.2, had 17 pairwise SNPs and with no AMR mechanisms detected, were susceptible to all drugs. Pangenome analysis of the two isolates found they shared 4,546/4,547 genes with the difference a hypothetical protein.

### Antimicrobial resistance profiles in S. Typhi.

**FIG 2:**
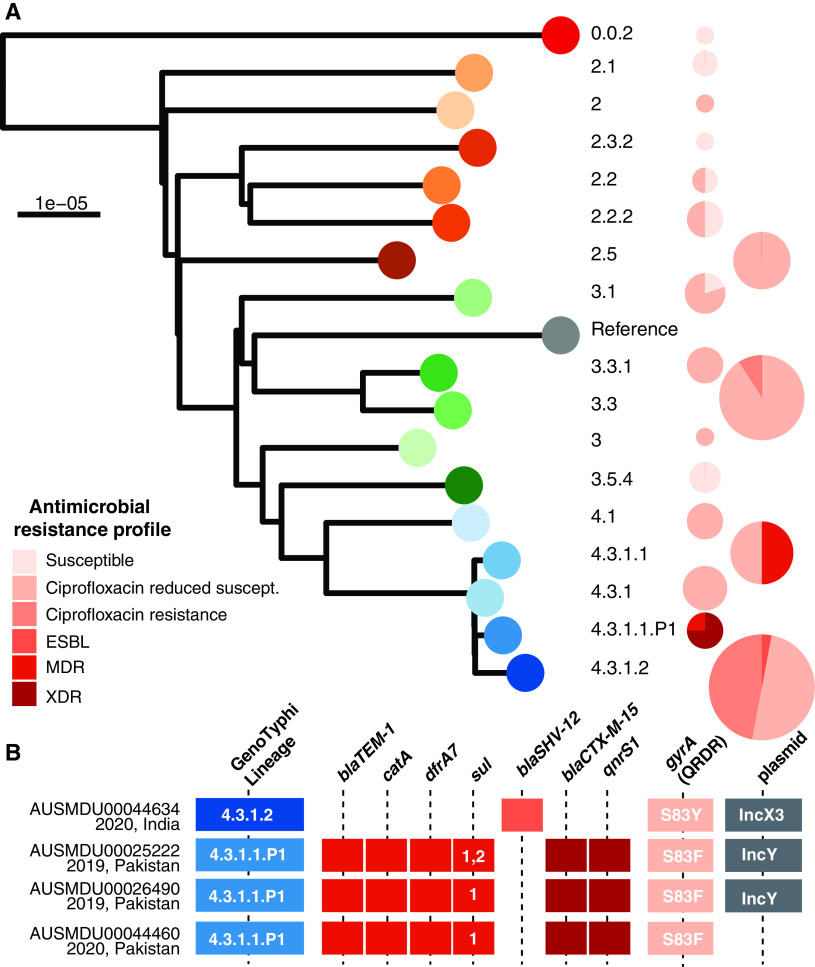
Antimicrobial resistance profiles in Salmonella enterica serovar Typhi (S. Typhi) data set. A: Framework tree of the 17 different GenoTyphi lineages identified, using reference genome CT18 (accession AL513382). Pie graphs to the right show the number is isolates in each lineage and are colored by the AMR profile: susceptible, reduced susceptibility to ciprofloxacin (1–2 point mutations in QRDRs and no other AMR mechanisms), ciprofloxacin resistant (3-point mutations in QRDRs), ESBL, MDR or XDR. B: Characterization of four S. Typhi isolates where ESBL genes were detected. The genotype membership, AMR determinants to therapeutic options are shown (with specific alleles given for *sul* genes and point mutations in QRDRs), as are the presence of plasmid replicons. AMR, antimicrobial resistance; QRDR, quinolone resistance determining region; XDR, extensively drug resistant; ESBL, extended spectrum beta-lactamases.

Different AMR profiles characterized the S. Typhi genotypes detected in the Australian cohort (Fig. 2, Fig. S1 in the supplemental material). A total of 10 out of 116 (8.6%) cases were either MDR (*n* = 7) or XDR (*n* = 3). The XDR isolates were genotyped as 4.3.1.1.P1, while the MDR isolates were either 4.3.1.1 (*n* = 6) or 4.3.1.1.P1 (*n* =1). No genes associated with carbapenem resistance were detected in any of the S. Typhi. Ciprofloxacin resistance resulting from triple point mutations in QRDR was detected in two lineages, 4.3.1.2 (16/34, 47.1%) and 3.3 (2/22, 9.1%).

Third-generation cephalosporin resistance mediated by ESBLs was rare in the 2-year cohort, detected in 4/116 (3.4%) of S. Typhi (Fig. 2). All four of these ESBL isolates were phenotypically resistant to cefotaxime and no other isolates were phenotypically resistant to this drug. The three XDR isolates in 4.3.1.1.P1 with reported travel to Pakistan all had the same ESBL gene, *bla*CTX-M-15, the acquired fluoroquinolone resistance gene, *qnrS1*, a single QRDR mutation, *gyrA-*S83F, and the MDR profile (Fig. 2B). The IncY replicon gene was detected in two of the three XDR isolates, AUSMDU00025222 and AUSMDU00026490. Subsequent alignment of the short-read data of the three XDR isolates to the IncY p60006 plasmid, reported by Klemm et al. (14) from the XDR S. Typhi outbreak in Pakistan in 2018, found AUSMDU00025222 and AUSMDU00026490 had > 95% alignment to the reference. In contrast, AUSMDU00044460 only had 55.7% alignment to the IncY plasmid and the absence of the IncY replicon gene, suggestive of integration into the chromosome as has been previously reported by Nair et al. (4). Inspection of assembly graphs in Bandage (27) was unable to confidently infer the integration from short read data alone. The AMR profile of AUSMDU00044460 is different to those previously characterized in integrating into the chromosome with the absence of genes mediating resistance to streptomycin and presence of *bla*TEM-1 (4, 8, 14).

The S. Typhi isolate AUSMDU00044634 had a unique ESBL profile in the Australian data. This isolate genotyped as 4.3.1.2 and was from a case with reported travel to India. Only a single AMR gene was detected, *bla*SHV-12, that mediates resistance to extended spectrum beta-lactams. AUSMDU00044634 also had a single point mutation detected, *gyrA-*S83Y, conferring reduced susceptibility to ciprofloxacin; and the IncX3 plasmid replicon. Both AUSMDU00044634 and the Klebsiella pneumoniae pIncX-SHV plasmid (the plasmid replicon reference sequence) had >92% alignment to the reference IncX plasmid, pLHST2018, from a S. Typhi strain collected in India in 2018 (28). The pLHST2018 plasmid also carries the *qnrB7* quinolone gene which was absent in the AUSMDU00044634 genome. Further, the IncX plasmid replicon and *bla*SHV-12 gene are rare in S. Typhi. This profile has only been reported in three S. Typhi isolates on TyphiNET; all from samples collected in India in 2016, although these three public isolates also had triple point mutations in QRDRs and the *qnrB* gene.

Point mutations known to confer resistance to either ciprofloxacin or azithromycin were screened for in all isolates. No known point mutations were detected for azithromycin resistance and no isolates were phenotypically resistant to azithromycin. Only 11 isolates had no point mutations in QRDRs, and with no other AMR determinants detected, were completely susceptible to all drugs (Table S1 in the supplemental material). These were found in isolates from cases with reported travel to Indonesia (*n* = 2), India (*n* =2), Cambodia (*n* = 1), Mexico (*n* =1), Samoa (*n* = 1), Pakistan (*n* =1), or no travel (*n* =3). None of these 11 isolates genotyped as part of the global clone (4.3.1 and related sublineages).

Nearly all isolates were either classed as resistant to ciprofloxacin (18/116; 15.5%) or to have reduced susceptibility to ciprofloxacin (87/116; 75.0%). The triple mutation profile of *gyrA-*S83F, *gyrA*-D87N and *parC-*S80I was associated with lineage 4.3.1.2 (16/34, 47.1%), whereas the profile *gyrA-*S83F, *gyrA*-D87V, and *parC-*S80I was associated with lineage 3.3 (2/22, 9.1%) (Fig. 2A, Table S1 in the supplemental material). Double point mutations were detected in three isolates all genotyped as 4.3.1.2, *gyrA-*S83F, *parC-*E84G (*n* = 2) and *gyrA-*S83Y, *parC-*E84G (*n* = 1). The remaining 84/116 (72.4%) genomes had a single mutation detected; the most common being *gyrA-*S83F in 65 isolates (Fig. 2, Fig. S1).

### High-risk AMR lineages in returned travelers.

**FIG 3:**
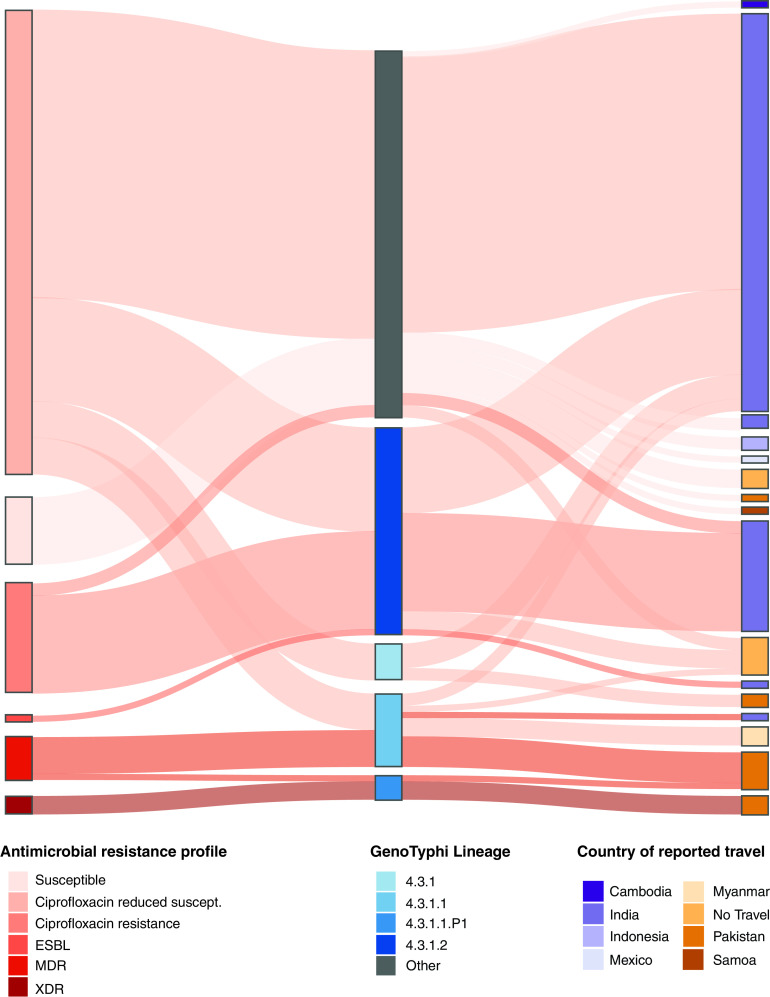
Relationship of AMR profiles with lineage and reported travel. Sankey diagram showing the relationships between three variables; nodes to the left, antimicrobial resistance profile to key treatment options defined by presence of genetic mechanisms resulting in XDR, MDR, ESBL resistance, ciprofloxacin resistance, ciprofloxacin reduced susceptibility or susceptible isolates; center nodes, membership to one of the global GenoTyphi lineages or other; and nodes to the right, country of reported travel. The connections between the nodes are colored by antimicrobial resistance profile. XDR, extensively drug resistant; MDR, multidrug resistance; ESBL, extended spectrum beta-lactamases.

Reported country of travel was a key marker for high-risk genotypes and AMR profiles (Fig. 3, Fig. S1A to S1D in the supplemental material). Three of the four most prevalent lineages in the Australian data were associated with AMR profile and country, 3.3 and 4.3.1.2 with ciprofloxacin resistance and travel to India, and 4.3.1.1 with MDR and travel to Pakistan. All isolates in the fourth most common lineage 2.5 had a single point mutation *gyrA*-S83F and 6/10 (60%) had the IncFIB plasmid replicon detected. The pangenomes of these four lineages was similar, with plasmids likely to be the key difference in accessory genome content within the lineages (Fig. S1E and Table S1 in the supplemental material). Detailed statistical analysis of stratified data based on genotype prevalence, country, and AMR profile was not able to be conducted due to small numbers in the Australian data. However, previously reported AMR patterns associated with genotype lineage and country of travel were found in the Australian S. Typhi cohort.

Triple mutations associated with ciprofloxacin resistance were found only in isolates from cases with reported travel to India. The main sublineage was 4.3.1.2 although genotype 3.3 also had isolates with triple QRDR mutations. This represents 20.7% of all cases with reported travel to India, noting that most had at least a single QRDR mutation. The 4.3.1.2 lineage was the most common genotype in the Australian cohort, with 47.1% having an AMR profile of ciprofloxacin resistant. The 4.3.1.2 lineage was most common in the TyphiNET data for Indian S. Typhi genomes from both local and travel-associated cases, with triple point mutations detected in 10.5 and 35.6%, respectively.

In contrast, travel to Pakistan was associated with XDR and MDR isolates of S. Typhi that were part of lineage 4.3.1.1.P1 and lineage 4.3.1.1 respectively. Thus, nine of 12 (75.0%) of all returned travelers from Pakistan were at least resistant to chloramphenicol, ampicillin, and co-trimoxazole, and for 25% of cases, the only remaining effective oral therapeutic was azithromycin. The high levels of AMR detected in the Australian data with travel to Pakistan was reflected in the S. Typhi genomes associated with Pakistan reported on TyphiNET, with 52.6% being typed as 4.3.1.1.P1 and XDR.

## DISCUSSION

In this study we undertook an unbiased 2-year cohort study of S. Typhi cases that demonstrated the value of enhanced regional surveillance provided through greater integration of epidemiological and genomic data. We show that high risk lineages, associated with MDR, XDR and ciprofloxacin resistance mechanisms, strongly correlate to country of reported travel. Ongoing integrated analysis will be critical with the prospect of increasing resistance to azithromycin, the last broadly effective oral therapeutic (22), and increasing cases of XDR S. Typhi that have been reported globally (9, 14, 24).

The two main travel destinations associated with Australian cases of typhoid fever are India and Pakistan in South Asia, and it is from this region that new AMR patterns in S. Typhi are largely emerging (9, 15, 23). The current therapeutic guidelines for typhoid fever in Australia recommend use of ceftriaxone or azithromycin for infections acquired in Southeast Asia or the Indian subcontinent in the first instance, and quinolones if confirmed as susceptible (29). We note the Australian data are biased by local travel patterns and that vaccination status of cases are not routinely collected (vaccination is recommended for travelers to typhoid endemic regions). Despite these limitations, these data demonstrate that enhanced genomic surveillance of vaccine preventable invasive bacterial pathogens provides opportunities for more targeted treatment guidelines in the future. For example, the ESBL resistant isolate of 4.3.1.2 is not susceptible to one of the two recommended drugs in Australia based upon travel history, but it is susceptible to most oral therapeutics including co-trimoxazole. Further, the relatively high rates of ESBL resistance in returned travelers from Pakistan, would suggest that a broader-spectrum antimicrobial, such as a carbapenem (for severe disease) or azithromycin, are more appropriate initial antimicrobials for these cases at present.

Notably, we detected known AMR profiles that have been previously found to be associated with different lineages and countries, including when the AMR profiles were rare and newly emerging (14, 15, 21, 28). This is best as exemplified by the characterization of the IncX plasmid with the *bla*SHV-12 gene detected in an isolate from an Australian case with recent travel to India, which was also identified from another isolate collected in India (28). It has only been reported in three isolates on TyphiNET and only a single S. Typhi genome in the recent 860 cases from Public Health England was also found to have the *bla*SHV-12 gene (15). This demonstrates the very early detection of a new AMR profile through regional surveillance. It is likely that this profile resulted from a plasmid acquisition event from another member of the *Enterobacterales* circulating in India; most likely K. pneumoniae, which most commonly carry *bla*SHV ESBL genes, usually on plasmids (30). As such, it is anticipated that additional plasmid acquisition events will continue to occur within lineages of S. Typhi. This has previously been suggested as the means of ESBL resistance for a recent S. Typhi isolate collected in the Demographic Republic of the Congo in 2015 that was genotyped as lineage 2.5.1 (31). This demonstrates the role for routine surveillance within public health laboratories to provide an early warning signal of potential new threats.

Importantly, while no carbapenemase resistance genes were detected in the Australian cohort, and have not been detected in S. Typhi at all to date, the potential prospect of MDR, azithromycin, or ciprofloxacin resistant S. Typhi acquiring a plasmid with carbapenemase genes would be dire. Particularly as resistance to ESBLs and azithromycin have both emerged in the past few years, highlighting the rapid pace at which S. Typhi is acquiring AMR mechanisms. Both Escherichia coli and K. pneumoniae are ubiquitous in the gastrointestinal tract and may harbor carbapenemase resistance plasmids (such as *bla*NDM-carrying E. coli, commonly detected in the community in South Asia) (32), and, given the right selective pressures, such plasmids would be retained upon acquisition. Detecting these new AMR profiles as they emerge, characterizing the underlying genetic mechanism and genotype, and linking these data to travel history will be critical for ongoing surveillance and response to S. Typhi and may inform public health and clinical practices.

The ongoing pandemic and coincident increase in antimicrobial therapy for patients with severe COVID-19 infections may escalate the levels of AMR in countries such as India, which has high incidence of COVID-19 (33–35), may represent a serious threat to public health both locally and globally. While noting that international travel will be limited in the near future as a result of the ongoing pandemic, efforts can be made to prepare the emergence of these threats. Internationally, Pathogenwatch (9, 36) and TyphiNET, have been developed to analyze and report on all public S. Typhi genomes, providing breakdowns of AMR and genotypes by country, and already are valuable resources. In Australia, the newly established AusTrakka platform (https://www.cdgn.org.au/austrakka) is the nationally recognized platform for real-time analysis of integrated pathogen genomic data for public health purposes. AusTrakka provides a central platform for the secure sharing of data at both within and between different state and territory jurisdictions, and S. Typhi will be included on the platform. As such, ongoing genomic surveillance efforts on integrated platforms both nationally and internationally will be key for S. Typhi.

This study provides a comprehensive baseline for future genomic surveillance of S. Typhi in Australia and the surrounding region. Integrating genomic and epidemiological data for prospective surveillance will ensure emerging drug-resistant S. Typhi threats are identified early, and treatment guidelines can be appropriately adjusted. Global efforts to address the ongoing threat of typhoid, and emerging drug-resistant clones, remain critically important.

## MATERIALS AND METHODS

### National surveillance for typhoid fever.

The National Notifiable Disease Surveillance System (NNDSS) was established in Australia in 1990. The NNDSS coordinates the surveillance for communicable diseases. Notifications of disease, such as typhoid fever, are made to the appropriate health authority in each jurisdiction in the federated nation and these data are then in turn supplied to the Australian Government of Health. The raw counts of typhoid fever by State and Territory and Year were obtained from http://www9.health.gov.au/cda/source/rpt_4.cfm on 6 January 2021.

### Study setting.

In Australia, typhoid fever is a notifiable disease and S. Typhi isolates in Victoria are forwarded from diagnostic laboratories to the Microbiological Diagnostic Unit Public Health Laboratory (MDU PHL), the bacterial public health reference laboratory for the state of Victoria, for further characterization. Since July 2018, all Salmonella isolates received at the MDU PHL have been subject for whole-genome sequencing (WGS). An unbiased sampling approach was taken to include all 116 S. Typhi isolates received between 1 July 2018 to 30 June 2020. International travel data was for individual cases was obtained from routine case interviews conducted by the Victorian Department of Health.

### Ethics.

Data were collected in accordance with the Victorian Public Health and Wellbeing Act 2008. Ethical approval was received from the University of Melbourne Human Research Ethics Committee (study number 1954615.3).

### Whole-genome sequencing and quality control.

The original sample received at MDU PHL was subcultured to a Nutrient Agar (NA) plate and streaked to achieve single colonies. The NA plate was then incubated 37°C for 18–24 hr. A single colony is harvested, using a 1 μl sterile inoculating loop, and is emulsified into 200 μl lysis buffer. The genomic DNA of 116 isolates was extracted from a single colony using a QIAsymphony™ DSP DNA Virus/Pathogen Kit (Qiagen) according to manufacturer’s instructions, and WGS was performed using Illumina NextSeq with 150 bp paired-end reads. Genomes had a phred score of 33 and a depth score of ≥50.

### Phylogenetic analysis of S. Typhi isolates.

The 116 S. Typhi genomes were mapped to the standard S. Typhi reference CT18 (NCBI accession: AL513382) using Snippy (v4.6.0) (https://github.com/tseemann/snippy) using a minimum fraction of 0.9 and a minimum coverage of 10 reads at each base. Phage and repeat regions were masked from the final alignment using coordinates available in Ingle et al. 2019 (6), filtered for recombination using Gubbins (v2.4.1) (37) and the final SNPs extracted with SNP-sites (38). A maximum likelihood (ML) phylogenetic tree was inferred using IQ-Tree (v1.6.12) (39) from the SNP alignment of 3,040 bases using a generalized time-reversible model + constant sites and rapid bootstrapping (40). The final tree was mid-point rooted with phangorn (v2.7.1) (41) and visualized with ggtree (v3.0.2) (42). Ape (v5.5) (43) was used to drop tips from the full tree to generate the framework tree.

S. Typhi isolates were genotyped using GenoTyphi (https://github.com/katholt/genotyphi) (7) using the vcf files from Snippy output. GenoTyphi assigns isolates into the established extended typing framework with the global lineage (associated with haplotype H58) further delineated into sublineages associated with MDR (4.3.1.1), ciprofloxacin resistance (4.3.1.2), and XDR (4.3.1.1.P1) S. Typhi (13, 44). GenoTyphi detects known point mutations in the QRDRs in *gyrA* and *parC* genes and also detects the known point mutations (R717Q and R717L) associated with reduced susceptibility to azithromycin in *acrB.* Isolates with 3-point mutations in QRDR regions were defined as ciprofloxacin resistant isolates.

### Genome assemblies and screening of accessory genomes.

S. Typhi genomes were assembled using SPAdes (v 3.14.1) (45). The genome assemblies of all isolates were screened for acquired AMR determinants using the AMRFinder (46) database (https://github.com/ncbi/amr/wiki/AMRFinder-database) as implemented in the abriTAMR tool (https://github.com/MDU-PHL/abritamr). Plasmid replicons were detected using the PlasmidFinder database (47) with ABRicate (https://github.com/tseemann/abricate) using a minimum identify of 90% and minimum coverage of 90%. Isolates were serotyped *in silico* with SISTR (48). Tidyverse (v1.3.1) (49) was used to wrangle the data and ggplot2 (v3.3.5) used to visualize the data.

### Determination of antimicrobial resistance profiles.

Isolates with resistance determinants to ampicillin, chloramphenicol, and co-trimoxazole were defined as MDR. Isolates that, in addition to the MDR profile, also had a gene conferring resistance to ESBLs, the presence of *qnrS1* and at least one QRDR point mutation were defined as XDR. The designation ESBL was for S. Typhi isolates where an ESBL resistance gene was detected and the absence of other mechanisms (known AMR genes, triple point mutations in QRDRs, or a single point mutation in *acrB*) that would result in resistance. Isolates were classed as ciprofloxacin resistant if 3-point mutations in QRDRs were detected, while 1- or 2-point mutations in QRDRs (where no other AMR mechanisms were detected) resulted in a reduced susceptibility to ciprofloxacin profile. To visualize the relationship of the AMR profile to membership to GenoTyphi global lineage and country of reported travel was visualized as a Sankey plot with networkD3 (v0.4).

### Exploration of accessory genome content.

Differences in the accessory genome of the genotype lineages with ≥10 isolates were explored with Panaroo (v1.2.7) (50). Briefly, the .gff files from the annotated genomes assemblies were used as input to panaroo using the strict clean-mode and default parameters.

The presence of IncY and IncX plasmids were investigated in more detail for the isolates where *bla*_CTX-M-15_ and *bla*_SHV-12_ were respectively detected. Three isolates, AUSMDU00025222, AUSMDU00026490 and AUSMDU00044460 were aligned to the IncY plasmid, p60006 (accession: LT906492) of a S. Typhi isolate collected in Pakistan (14). AUSMDU00044634 was aligned to IncX plasmid, pLHST2018 (accession: CP052768), of a S. Typhi isolate collected in India (28) using snippy (v4.6.0). The publicly available pIncX-SHV (accession: JN247852) from K. pneumoniae collected in Italy (30) was also aligned using the –ctgs option in snippy.

### Investigation of infection linked to chronic carrier.

One isolate, AUSMDU00019653, was epidemiologically linked to a chronic case reported outside the 2-year cohort. The relatedness of AUSMDU00019653 to the isolate from the chronic case, AUSMDU00017205, was investigated first through mapping-based approaches to reference CT18 described above with pairwise SNP-distances determined with snp-dists (v0.7.0) (https://github.com/tseemann/snp-dists). AUSMDU00017205 was assembled as above and the pangenome of the two genomes was explored with Panaroo (v1.2.7) (50). AUSMDU00017205 was characterized for known AMR mechanisms and GenoTyphi lineage as above.

### Comparison of Australian S. Typhi with public data on TyphiNET.

Details on publicly available S. Typhi data that have been characterized for GenoTyphi lineage, AMR mechanisms and are accompanied by geographical data were downloaded from TyphiNET on 10th August 2021. The most frequent genotypes from India (the most common destination for travelers in the Australian data), prevalence of the XDR profile with genotype and country, and combination of IncX and *bla*SHV-12 were compared with the Australian data.

### Cefotaxime and azithromycin susceptibility testing.

Antimicrobial susceptibility testing for cefotaxime and azithromycin were performed for all isolates using agar dilution. Clinical and Laboratory Standards Institute (CLSI) 2019 breakpoints were used for interpretation. Isolates with a MIC ≥4 μg/ml defined as cefotaxime resistant. Isolates with a MIC ≥32 μg/ml defined as azithromycin resistant.

### Data availability.

Details and the accession numbers of the sequence data of genomes included in our analysis are available in Table S1 in the supplemental material, and the reads of isolates sequenced at MDU PHL are available on the NCBI Sequence Read Archive (BioProject PRJNA319593).
