# Supplementary material for: Genomic Epidemiology and Antimicrobial Resistance Mechanisms of Imported Typhoid in Australia
Source: Antimicrob Agents Chemother. 2021 Nov 17;65(12):e01200-21. doi: 10.1128/AAC.01200-21 (PMC8597785; doi:10.1128/AAC.01200-21)
Supplement: Supplemental file 1 — Supplemental material. Download aac.01200-21-s0002.pdf, PDF file, 0.1 MB [file aac.01200-21-s0002.pdf]

Supplementary Tables and Figures

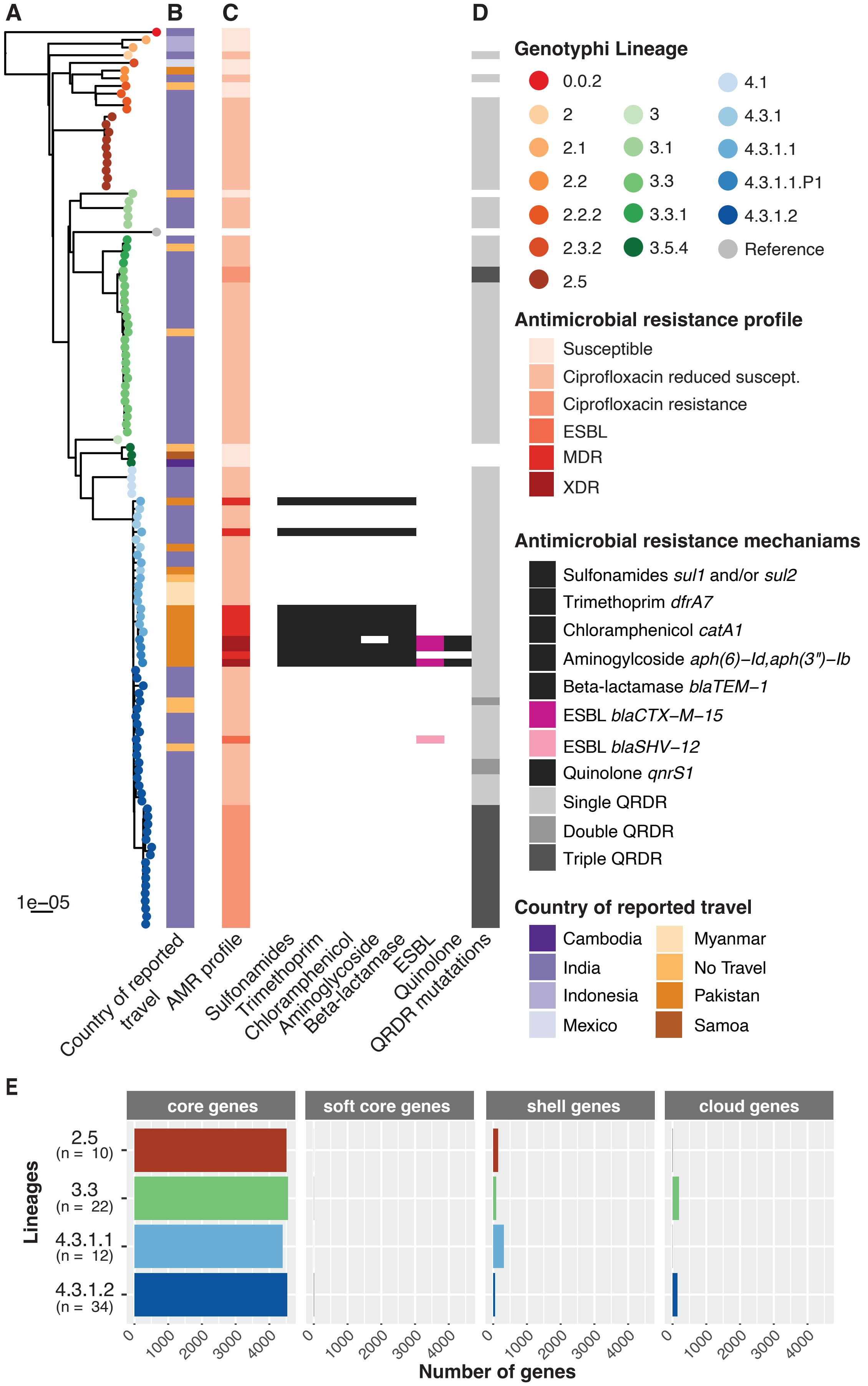

Supplementary Figure 1: Overview of the Australian *S.* Typhi two-year cohort

A) A maximum likelihood phylogeny of the 116 *S.* Typhi received at MDU PHL between 1st July 2018 and 30th June 2020. The tips of the tree are coloured by Genotyphi lineage. B) The country of reported travel. C) AMR profile and presence of individual genes associated with AMR and D) the number of point mutations in QRDRs are shown to the right of the phylogeny. E) Characterisation of pangenome content with the four GenoTyphi lineages with ≥10 isolates. Core gene were found in ≥99% of genomes, soft core genes in ≥95 to <99% of genomes, shell genes in ≥15% to <95% of genomes and cloud genes in <15% of genomes. MDU PHL: Microbiological Diagnostic Unit Public Health; AMR: antimicrobial resistance; QRDRs: quinolone resistance determining regions.

Supplementary Table 1: Summary of 116 *Salmonella* Typhi isolates in the two-year Australian cohort
